# *Aspergillus tamarii* mediated green synthesis of magnetic chitosan beads for sustainable remediation of wastewater contaminants

**DOI:** 10.1038/s41598-022-13534-1

**Published:** 2022-06-13

**Authors:** Reyad M. El-Sharkawy, Mahmoud A. Swelim, Ghada B. Hamdy

**Affiliations:** Botany and Microbiology Department, Faculty of Science, Benha University, Benha, 13511 Egypt

**Keywords:** Biotechnology, Microbiology

## Abstract

The release of different hazardous substances into the water bodies during the industrial and textile processing stages is a serious problem in recent decades. This study focuses on the potentiality of Fe_3_O_4_-NPs-based polymer in sustainable bioremediation of toxic substances from contaminated water. The biosynthesis of Fe_3_O_4_-NPs by *A. tamarii* was performed for the first time. The effect of different independent variables on the Fe_3_O_4_-NPs production were optimized using Plackett–Burman design and central composite design (CCD) of Response Surface Methodology. The optimum Fe_3_O_4_-NPs production was determined using incubation period (24 h), temperature (30 °C), pH (12), stirring speed (100 rpm) and stirring time (1 h). The incorporation of Fe_3_O_4_-NPs into chitosan beads was successfully performed using sol–gel method. The modified nanocomposite exhibited remarkable removal capability with improved stability and regeneration, compared to control beads. The optimal decolorization was 94.7% at 1.5 g/l after 90 min of treatment process. The reusability of biosorbent beads displayed 75.35% decolorization after the 7th cycle. The results showed a highly significant reduction of physico-chemical parameters (pH, TDS, TSS, COD, EC, and PO_4_) of contaminated wastewater. The sorption trials marked Fe_3_O_4_-NPs-based biopolymer as efficient and sustainable biosorbent for the elimination of hazardous toxic pollutants of wastewater in a high-speed rate.

## Introduction

Water has been deemed as one major constituent for the life sustainability on the earth^1–3^. The water deficiency problem is a hot issue facing both developing and developed countries. Water contamination due to industrialization, human activities, agricultural runoffs and overpopulation growth has serious harmful effect on human health and aquatic life^4,5^. Among various industrial pollutants, textile and industrial discharge are major serious contaminants in water bodies due to its non-degradable nature, mobility and high toxicity^6,7^. 

Textile dye contaminants can originate from various industries like paper, textile, plastic and tannery industries^5,8^. Such effluents are characterized by their high biological and chemical oxygen demand (BOD and COD), pH, total dissolved solids (TDS), organic residues, heavy metals, phosphates, sulfates, nitrates^2,4,6^. It is predicted that the industrial and municipal discharges will increase up to around 8 billion m^3^ in Egypt by 2030^9^. The treatment of these synthetic/toxic dyes before discharging into water bodies is highly desirable^4,8^. However, the elimination of these recalcitrant toxic dyes is remarkably difficult due to their chemical and physical properties.

Numerous chemical and physical technologies have been utilized like adsorption, precipitation, coagulation, and photocatalytic degradation^4,10–12^. Biological methods such as bioreactor, activated sludge system, biosorption and trickling filter have been employed for the treatment of textile and industrial wastewater^1,4^. However, these technologies have high operational costs, high-energy demand, production of hazardous toxic chemicals and design difficulty at large scale^8,13^. The development of an innovative eco-friendly and cost-effective strategy for the treatment of wastewater is highly desirable.

Among the existing technologies, adsorption is the most employed approach due to its simplicity, cheapness, reusability and effectiveness^4,5,14–16^. Adsorbent with large surface area, high adsorptive capacity, simplicity in operation, high reusability and regeneration are of low cost and great potentiality in the polluted wastewater treatment^8^. In this context, bioremediation of wastewater using metal oxide nanoparticles (NPs) has attracted significant attention. Of which magnetic nanoparticles have been mostly employed in the remediation processes as an alternative approach for the present expensive ones due to their magnetic character, high affinity, ecofriendly and cost effective^1,5,8^. Different approaches have emerged for the fabrication of various NPs including physical and chemical methods; however, biological synthesis of NPs using biomolecules produced by fungi, bacteria, actinomycetes and plants as stabilizing and capping agents have been utilized as effective tool for the synthesis of alternative adsorbents^11,17,18^.

The eradication of textile and industrial wastewater using alternative adsorbents, particularly synthetic and natural polymers, is of great interest^1,5,19^. Chitosan, a linear positively charged polysaccharides obtained by the deacetylation of chitin, has been used as effective adsorbent for toxic dyes^8,20^ and heavy metals^1,21^ due to its promising properties such as ecofriendly, bioactivity, renewability, nontoxic and high amino and hydroxyl contents^1,5,8^. In contrast, low surface area, porosity, and stability in acid medium are the main impediments for broad applications of chitosan. To overcome such shortcomings, chitosan has engaged with other material, particularly magnetic nanoparticles, which are characterized by well dispersed into polymer matrix^4,5,8,22^. The integration of magnetic nanoparticles into beads of chitosan, creates mechanically stable beads even in harsh conditions, performs porous beads instead of nonporous ones, augments the surface area, ability for surface modifiability, and can improve the biosorption potentiality of the as-prepared beads^1,5,8^. Magnetic-chitosan based gel has employed as promising adsorbent of various pollutants in aqueous solutions.

The specific objectives and novelty of the current research were to facile myco-synthesis Fe_3_O_4_-NPs/Chitosan beads and its application in sustainable decontamination of wastewater in very short period with considerable rapid removal capacity and high removal rate. The purpose of the study was accomplished via biosynthesis of Fe_3_O_4_-NPs using the metabolites of *A. tamarii*, optimize the biosynthetic process using Response Surface Methodology, synthesis of encapsulated magnetic-chitosan gel beads (MchiBs) without using any cross-linkers, assess and evaluate the potentiality, sustainability and stability of MchiBs in the treatment of textile dyes and determine the physicochemical properties of the textile and industrial wastewater after MchiBs treatment.

## Results and discussion

### Genetic confirmation of the fungal strain identity

**Figure 1:**
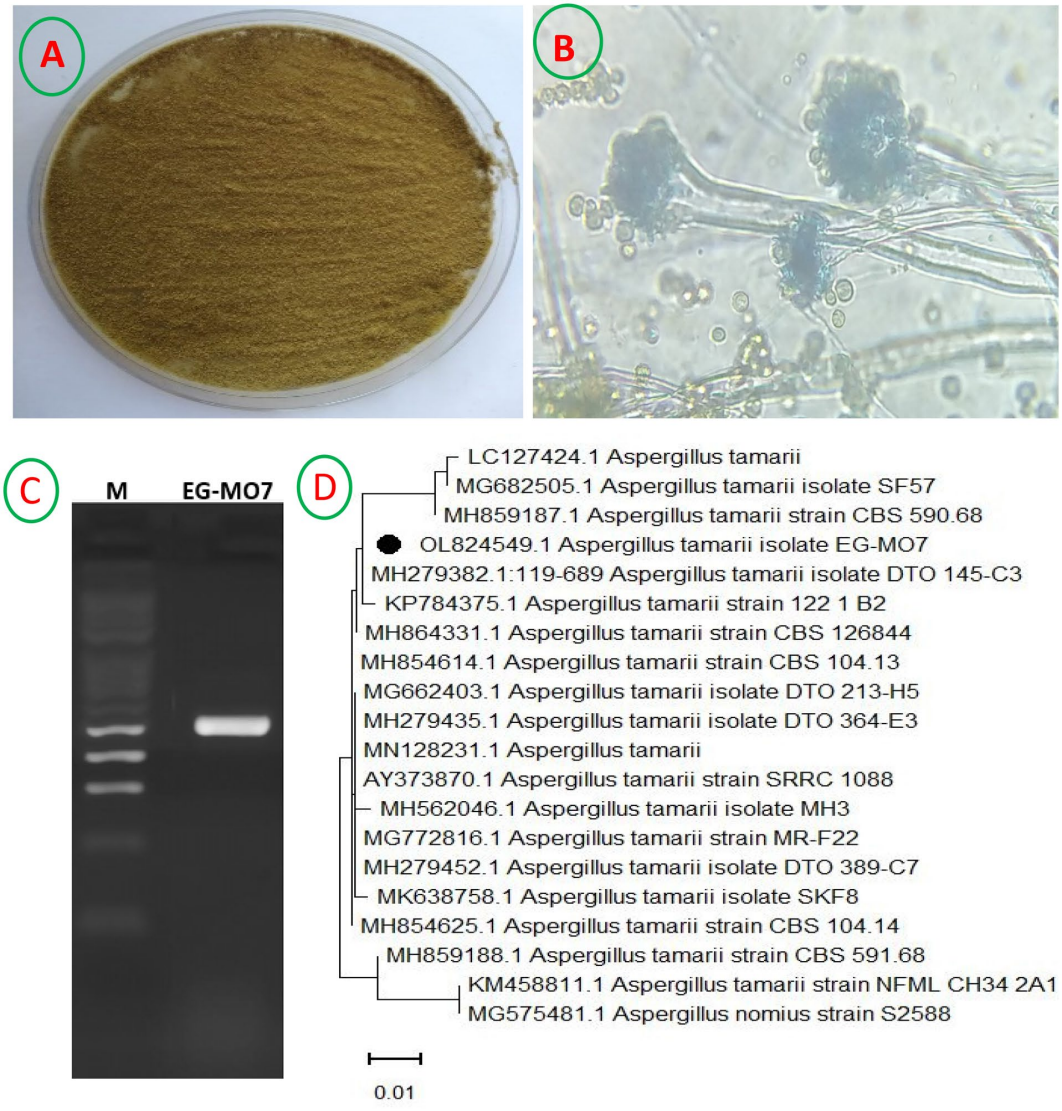
(**A**) Macro- and (**B**) Micro-morphology of *Aspergillus tamarii* grown on Czapek’s-Dox agar at 30 °C for 5 days. (**C**) PCR amplicons of the fungal strain EG-MO7 using the primers ITS1 and ITS4 (Uncropped gel image in Fig. 1 is in the supplementary information Fig. S1). (**D**) Phylogenetic analysis of *A. tamarii* and reference sequences conducted with MEGA-X 11 software based on the Neighbor-Joining method. The isolate in the present work refers by Filled black circle symbol.

The purified fungal strain EG-MO7 was morphologically and microscopically identified as *Aspergillus* sp. (Fig. 1A,B), based on the identification key^23^. The morphological identity was further ascertained based on its sequence of ITS fragments. The size of PCR amplicons was found to be around 571 bp (Fig. 1C, uncropped gel image of PCR product in Fig. S1). In the NCBI database, the non-redundant BLAST search revealed that the fungal strain EG-MO7 was closely related to *Aspergillus tamari* and its ITS sequence was deposited in the database of NCBI with accession number: OL824549.1. The phylogenetic tree of such ITS sequence was constructed via the Neighbor-joining method using a confidence level of 1000 bootstrap (Fig. 1D). The *A. tamarii* OL824549.1 displayed a similarity percentage of 99% with *A. tamarii* retrieved database deposited isolates MH2793821.1, KP784375.1, MH859187, and MN128231.1 with E-value of 0.0 and query coverage of 100%.

### Optimization of process factors for Fe_3_O_4_-NPs production

The effects of five independent variables on the process of magnetic nanoparticles production were screened using the two factorial Plackett–Burman design, which is important for reducing the number of repetitive trials during process optimization^24^. The values of the maximum and minimum level of the input parameters and the multiple-regression analysis of the process are shown in Table 1. The regression equation from PBD, after omitting the non-significant factors (*P* > 0.05), was as the following

1 $$Y = 4.31 - 0.0425\,X_{1} + 0.1533\,X_{3} - 0.727\,X_{5}$$

**Table 1:** ANOVA analysis of Plackett–Burman design for the optimization of process variables.

Variable code	Variable name	Level		*F*-value	*P*-value
		−1	+ 1		
X_1_	Incubation period	24 h	48 h	15.02	0.008*
X_2_	Temperature	25 °C	30 °C	0.02	0.903
X_3_	pH	5	12	16.63	0.007*
X_4_	Stirring speed	100	150	0.13	0.726
X_5_	Stirring time	1 h	2 h	7.62	0.033*

**Figure 2:**
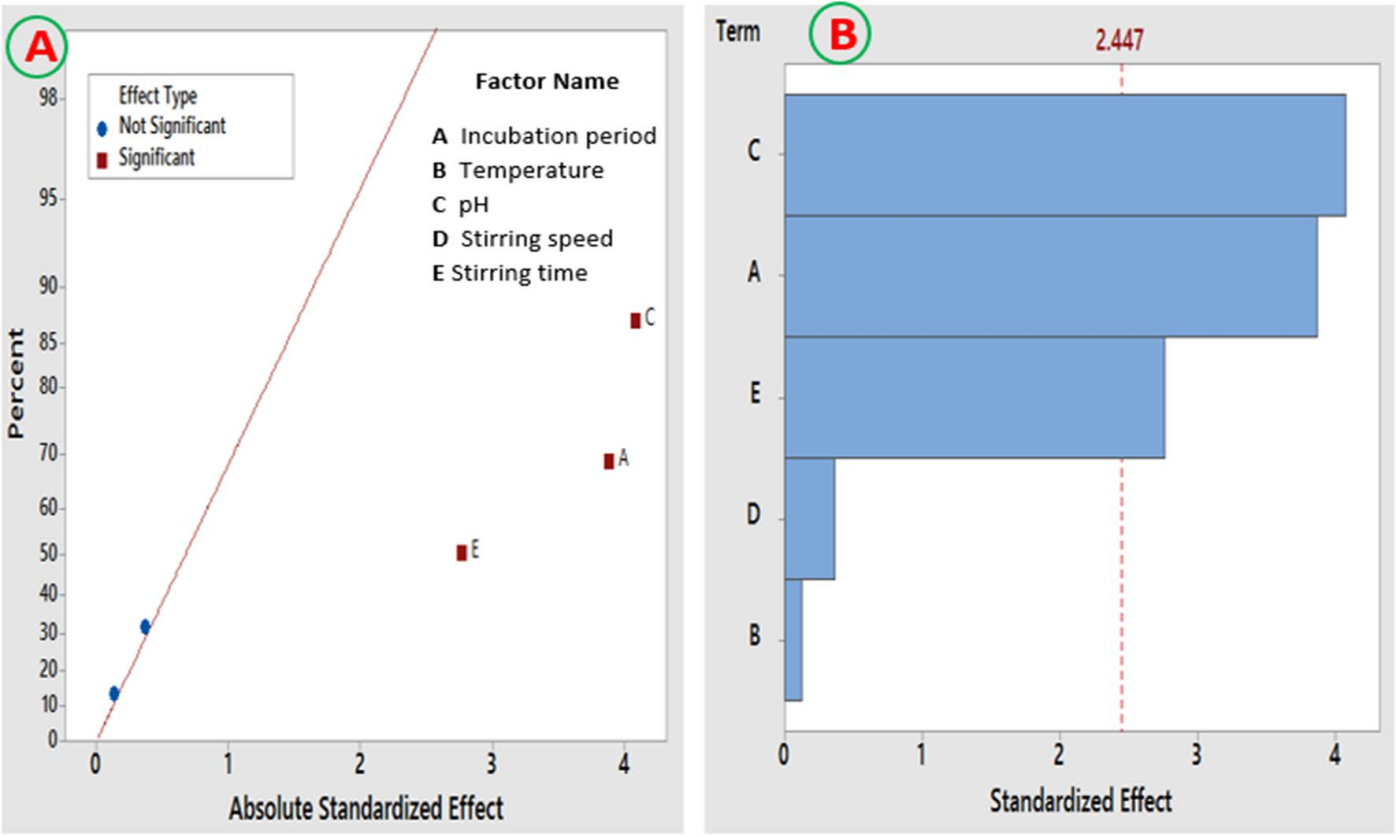
Main effects of the various investigated variables on the Fe_3_O_4_-NPs production by *A. tamarii* using Placket–Burman design. (**A**) The normal plot of the standardized effects for the Fe_3_O_4_-NPs production based on the first-order equation and (**B**) Pareto-chart of standardized effects illustrates the significance of each variable on the Fe_3_O_4_-NPs production process. Any variable pass beyond the red line is reported as statically significant.

The optimum magnetic nanoparticle production by harnessing the biomolecules of *A. tamarii* was recorded at the trial number 2, with the contribution of incubation period (24 h, −1), temperature (30 °C, + 1), pH (12, + 1), stirring speed (100 rpm, −1) and stirring time (1 h, −1). A minimum production of the myco-synthesized magnetic nanoparticle was detected in the 1st run. The highest effect (87.04%) on the production process was recorded for the input factor (pH, X_3_) as shown in Fig. 2A, followed by the incubation period (X_1_) and the stirring time (X_5_). A negative sign indicates antagonistic effect, whereas a positive one represents a synergistic effect. The main effects of the investigated variables on Fe_3_O_4_-NPs production using PBD was graphically illustrated as a Pareto chart in Fig. 2B. The main influential variables affecting the nanoparticle production process was optimized by the application of response surface methodology (RSM) using CCD analyses.

**Figure 3:**
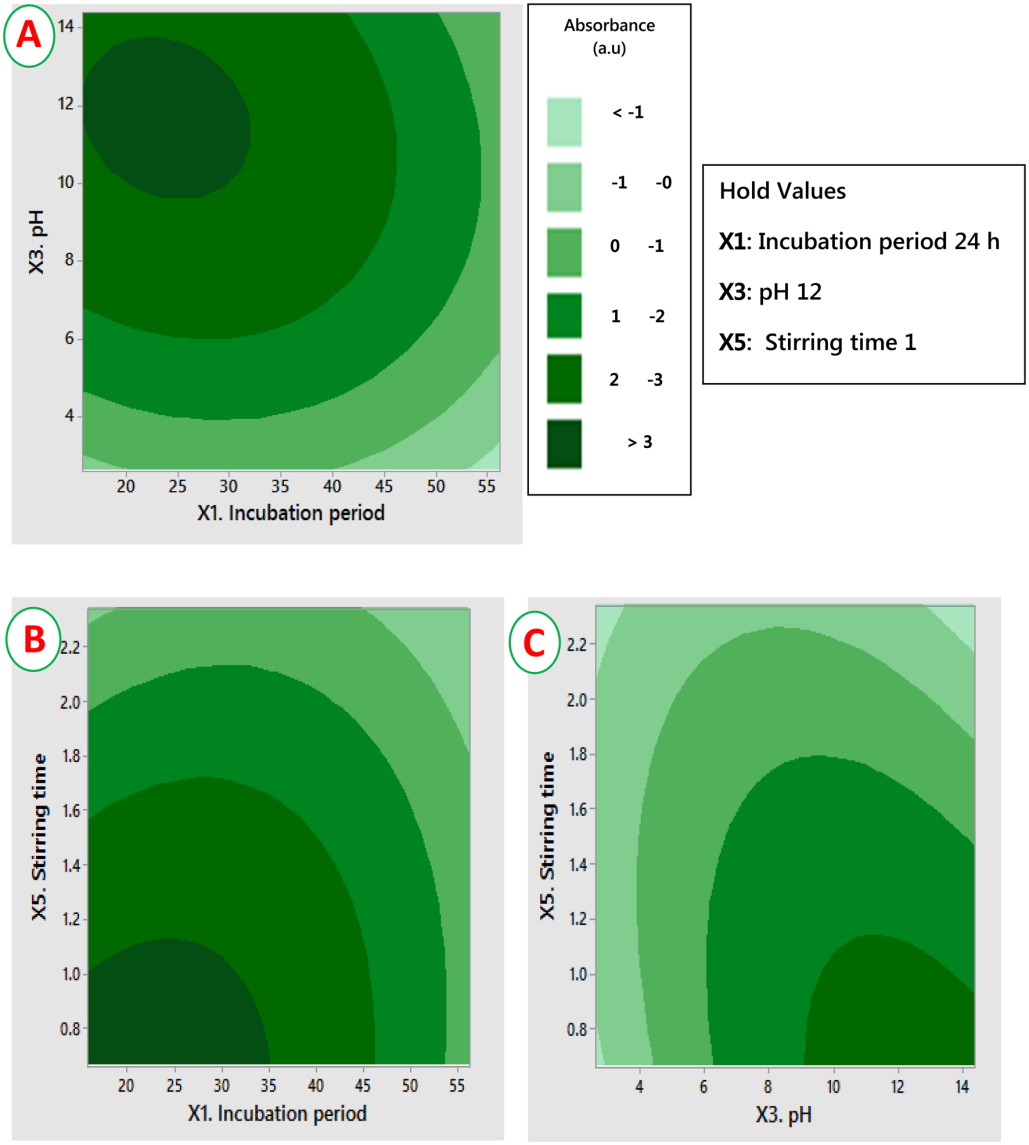
Contour response surface graphs illustrating the interactive effect among (X_1_) incubation period, (X_3_) pH, and stirring time (X_5_) on the Fe_3_O_4_-NPs production by harnessing the exo-metabolites of *A. tamarii*.

The three remarkable factors with positive effect on the dependent variable (response) were applied in CCD as central values. The significance of the RSM model was assessed by ANOVA and the results are briefly listed in Table 2. The results shown that the significant model parameters (*P* < 0.05) are X_1_, X_3_, X_5_, and X_3_ *X_5_. The interactive effects among two independent factors, when the third factor is at optimal level, were presented by 2D-contour plots (Fig. 3A–C). The 2D plots demonstrated that the production of nanoparticles was significantly increased in the middle value for each factor; however, the production process was significantly reduced by the variation in the factors levels, beyond the optimum level.

**Table 2:** ANOVA table for the impact of incubation period, pH, and stirring time on the magnetic nanoparticles production using the metabolites of *A. tamarii*.

Variable code	Sum of squares	*F*-value	*P*-value
X_1_	2.0890	13.19	0.005
X_3_	1.6275	10.27	0.009
X_5_	2.5438	16.06	0.002
X_1_^2^	1.6818	10.62	0.009
X_3_^2^	2.9571	18.67	0.002
X_5_^2^	0.5776	3.65	0.085
X_1_ X_1_	0.1540	0.97	0.347
X_1_ X_5_	0.2415	1.52	0.245
X_3_ X_5_	0.9045	5.71	0.038

The biosynthesis of magnetic nanoparticles is of great significant in the biomedical and biotechnological applications. Different physic-chemical properties are remarkably affected the biosynthesis process. The optimization of the influential factors is significantly desired. PBD and RSM are considered as one of the important statistical analysis which provides adequate information on the significance of real variables in the Fe_3_O_4_-NPs^25,26^. Such techniques are applied for the determination of the major variables affecting the process and the interactive connections among the output and input parameters^25^. The significant influences of temperature, stirring time and pH have been reported by other researchers^26,27^.

The results displayed a maximum peak height of 3.68 (Table S1), which was incredibly analogous to the predicted value by using CCD analysis and remarkably close to the best trial (Run no. 3). The experimental and predicted values were found to be satisfactorily correlated, indicating that the relationships between the investigated variables and the Fe_3_O_4_-NP_s_ biosynthesized by *A. tamari* can adequately described by the empirical model derived from CCD. Therefore, such model could be employed for the prediction of the biosynthesis of Fe_3_O_4_-NP_s_. Several researchers were approved the exo metabolites of fungai, bacteria, actinomycetes and plants as stabilizing and capping agents for the green synthesis of various nanoparticles^17,28,29^.

## Characterization

### UV–vis spectra, FT-IR and TEM analyses

**Figure 4:**
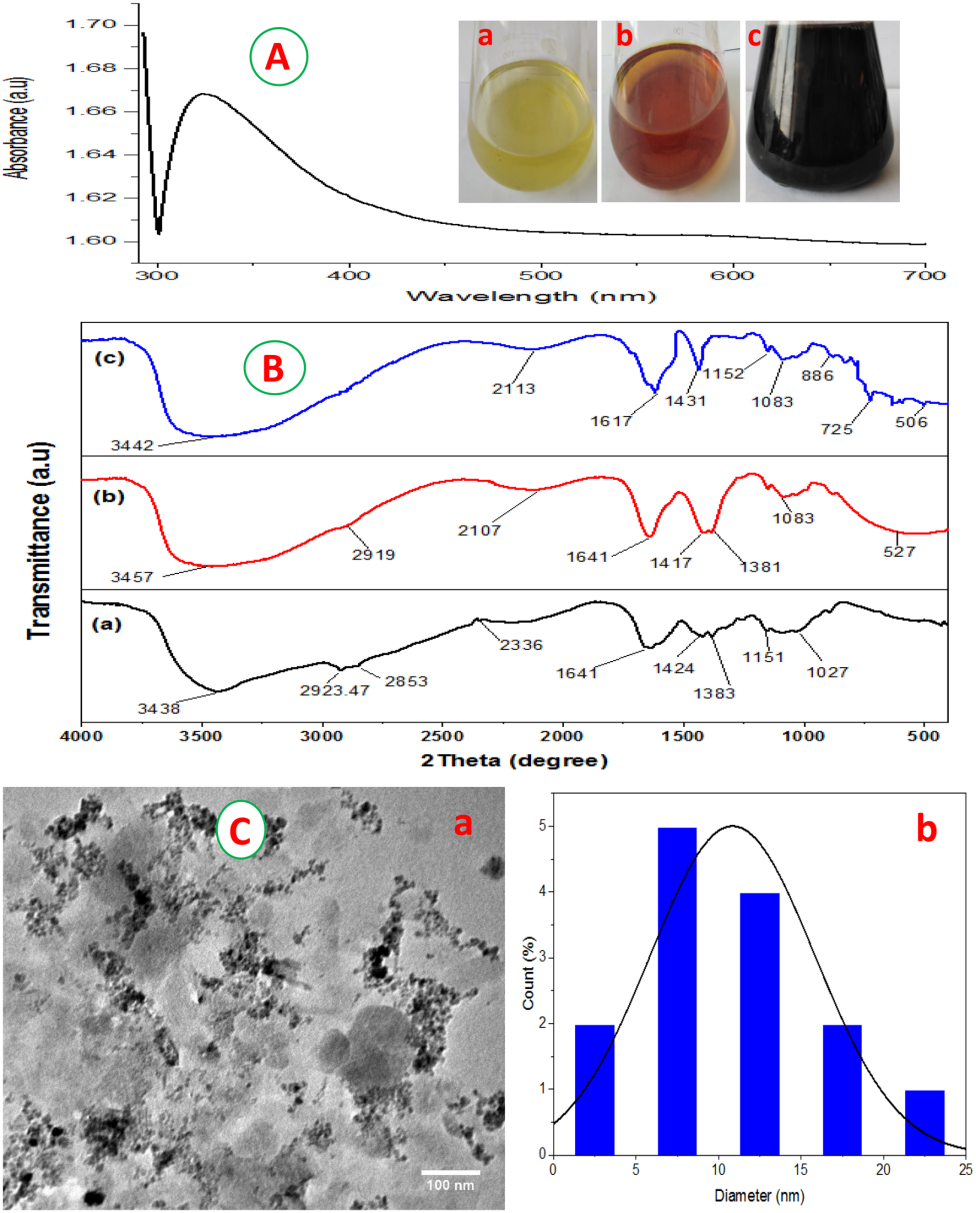
(**A**) UV–visible spectra at 290–700 nm of indigenous Fe_3_O_4_-NPs obtained by harnessing metabolites of *A. tamari* EG-MO7; inset shows (**a**) FeSO_4_.7H_2_O, (**b**) FeCl_3_.6H_2_O, and (**c**) the biosynthetic nanoparticles; (**B**) FT-IR spectrum of (**a**) chitosan (**b**) Magnetic chitosan beads (**c**) after dye adsorption onto magnetic chitosan beads; (**C**) Transmission electron microscope (TEM) picture of Fe_3_O_4_-NPs with a scale bar of 100 nm (**a**) Histogram presenting the size distribution of nanoparticles based on the TEM picture using Image J software portal (**b**).

The capability of the fungal mycelial exo-metabolites for the formation of biosynthetic Fe_3_O_4_-NP_s_ was initially observed by the color transformation of reaction mixture to deep black (Fig. 4A(a–c)). The formation of magnetic nanoparticles was then ascertained by UV–visible spectra. A characteristic absorption peak at 325 nm, which is corresponding to magnetite nanomaterial was observed (Fig. 4A) in agreement with^30–32^. The color change is probably attributed to the presence of various biomolecules in fungal extract, which delivers the reduction of different metal ions and the surface plasmon resonance of metal oxide. Meanwhile, the biosynthesis of Fe_3_O_4_-NPs appropriately stabilized under alkaline environment by the means of these biomolecules^4,33^.

The FT-IR analysis was used for detecting the prosperous incorporation of Fe_3_O_4_-NPs into chitosan beads and the potentiality of dye adsorption using MchiBs (Fig. 4B(a–c)). The peak at 3438 cm^-1^ could attribute to the N–H and O–H overlapping stretching vibrations^28^. The absorption bands observed at 2923 cm^-1^ and 2853 cm^-1^ could respectively ascribed to the O–H and C–H stretching vibrations of alcohol, carboxylic and alkane groups. Two peaks at 1383 and 1027 cm^-1^ are corresponding to C–N and C–O stretching bands, respectively. A peaks at 1641 and 1148 cm^-1^ were assigned, respectively, to C=O stretching vibration of amide group and C–O–C of glycosidic bridge. In the MchiBs spectrum, a very low intensity peak at 1083 was determined which is related to Fe–OH stretching vibration. The peak at 527 cm^-1^ related to the stretching band of Fe–O group. Reactive red 198 selected for studying the decolorization capability using the MchiBs (Fig. 4c). The appearance of some new peaks at a range of 400–700 cm^-1^ may attributed to the successful adsorption of dye onto the chitosan- Fe_3_O_4_-NPs beads. The peaks become generally broad with slight shift and the intensity decreased after the sorption of dye onto MchiBs. Overall, the FT-IR spectra ascertained the incorporation of Fe_3_O_4_ nanoparticles into chitosan beads and the successful biosorption of dye by MchiBs. Similar results have reported by other researchers^1,4,34,35^.

The average size of the nanomaterial is one of the major parameters, evidently related to its activities^35,36^. Herein, the size and shape of Fe_3_O_4_-NPs were determined using TEM. The TEM image showed that the average size of the Fe_3_O_4_-NPs were from 5 to 22 nm with homogenous distribution and spherical morphology (Fig. 4C(a, b))^8^ observed that the biosynthesized Fe_3_O_4_-NPs had uniform spherical shapes with homogenous distribution. The potentiality of the metabolites produced by *A. niger* for the green synthesis of Fe_2_O_3_-NPs was confirmed by^37^.

### SEM and EDX analyses

**Figure 5:**
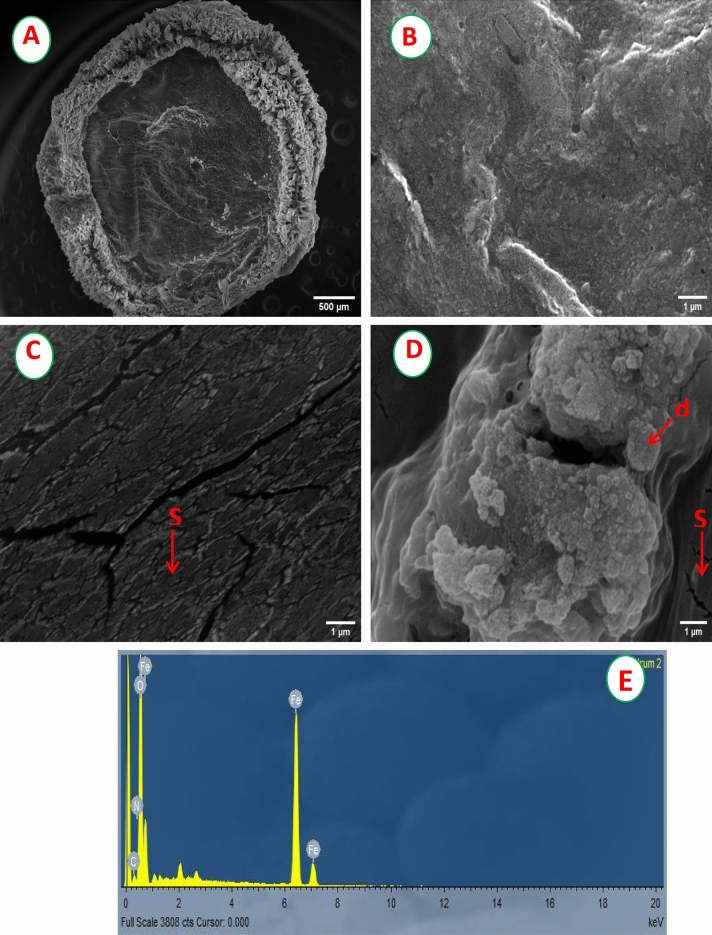
SEM pictures of (**A**) CchiBs at amplification of 500 µm, (**B**) highly magnified CchiBs, (**C**) MchiBs and (**D**) MchiBs after dye biosorption at amplification of 1 µm. EDX spectrum of MchiBs. S: Surface of MChiBs; d: Dye particles adsorbed on the surface of nanoparticles.

The digital images of control chitosan beads (CchiBs) and MchiBs have illustrated in (Fig. S2A-D). After the incorporation of magnetic nanoparticles, it’s observed that the color of CchiBs changed from white to whitish-black or grayish-white. The textural and surface morphology of both CchiBs (Fig. 5A,B) and MchiBs (Fig. 5C) were examined by SEM. The SEM images showed spherical irregular surface and porous structure. The surface area was increased by incorporating Fe_3_O_4_-NPs. The SEM pictures of the CchiBs and MchiBs before adsorption showed quite smooth surface with small grains and high porosity; however, the surface of MchiBs loaded with reactive red 198 as a model dye, shows the formation of aggregated dye particles, which were interconnected to each other (Fig. 5D). Several researchers stated that the behavior and biosorption efficiency is mainly dependent on the surface area as well as porosity of the nano-sorbent particles^10,19,38–40^. It is evident from the elemental analysis that the presence of Fe, O and N peaks in the EDX, indicating the fabrication of Fe_3_O_4_-NPs impregnated chitosan beads (Fig. 5E). The atomic percentages were C (11.76%), O (63.22%), N (7.30%) and Fe (17.72%) as detected by EDX.

### XRD analysis

**Figure 6:**
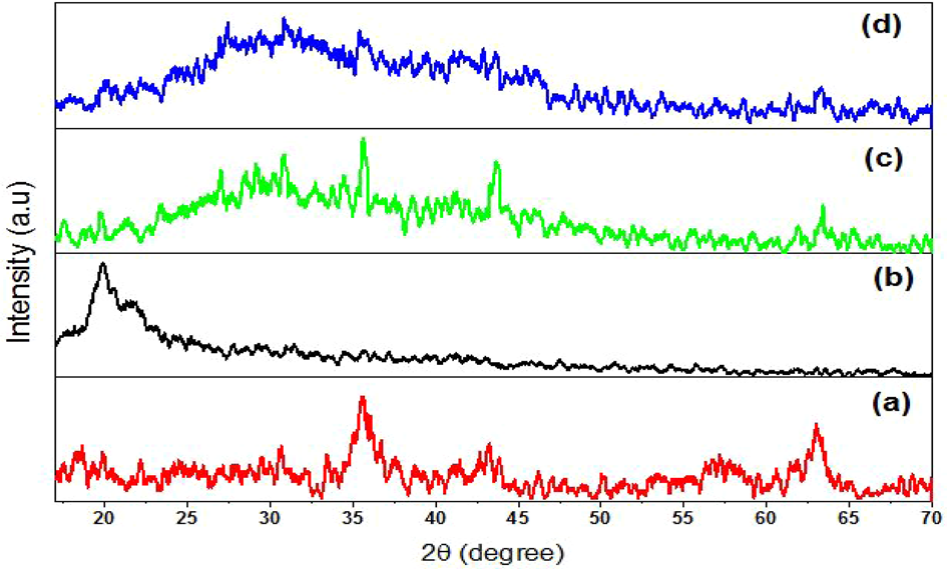
XRD pattern of Fe_3_O_4_-NPs (**a**), chitosan (**b**), MchiBs (**c**) and MchiBs after dye biosorption (**d**).

The XRD spectra of the biosynthesized Fe_3_O_4_-NPs clearly illustrates in Fig. 6a-d. Six diffraction peaks were observed at 2θ degree of 30.6°, 35.5°, 43.2°, 50.5°, 57.3°, and 63.01° which could attributed to (220), (311), (400), (422), (511), and (440), respectively. The appeared XRD peaks are matched to JCPDS card 01-089-3854^41^. The average size of Fe_3_O_4_-NPs crystal was found to be 16.5 nm using Debye–Scherrer Eq. ^42^. The XRD pattern of chitosan appeared as one broad peak at 19.87°^43^ confirm the amorphous structure of chitosan as the occurrence of broad peak at 20.1°. In the MchiBs spectrum, all-characteristic peaks of pure magnetite and chitosan appeared in Fig. 6c, hinting the incorporation of Fe_3_O_4_-NPs in the chitosan beads and no phase change in the magnetic nanocomposite. The peak intensity of chitosan and Fe_3_O_4_-NPs remarkably reduced after the formation of magnetic-chitosan beads. No noticeable changes in the XRD pattern have observed after the run of dye adsorption process; however, there is remarkable reduction in the peak intensities and appearance of new peaks. These findings agree with those of^1,35,44^.

### Treatment of textile wastewater using MchiBs

**Figure 7:**
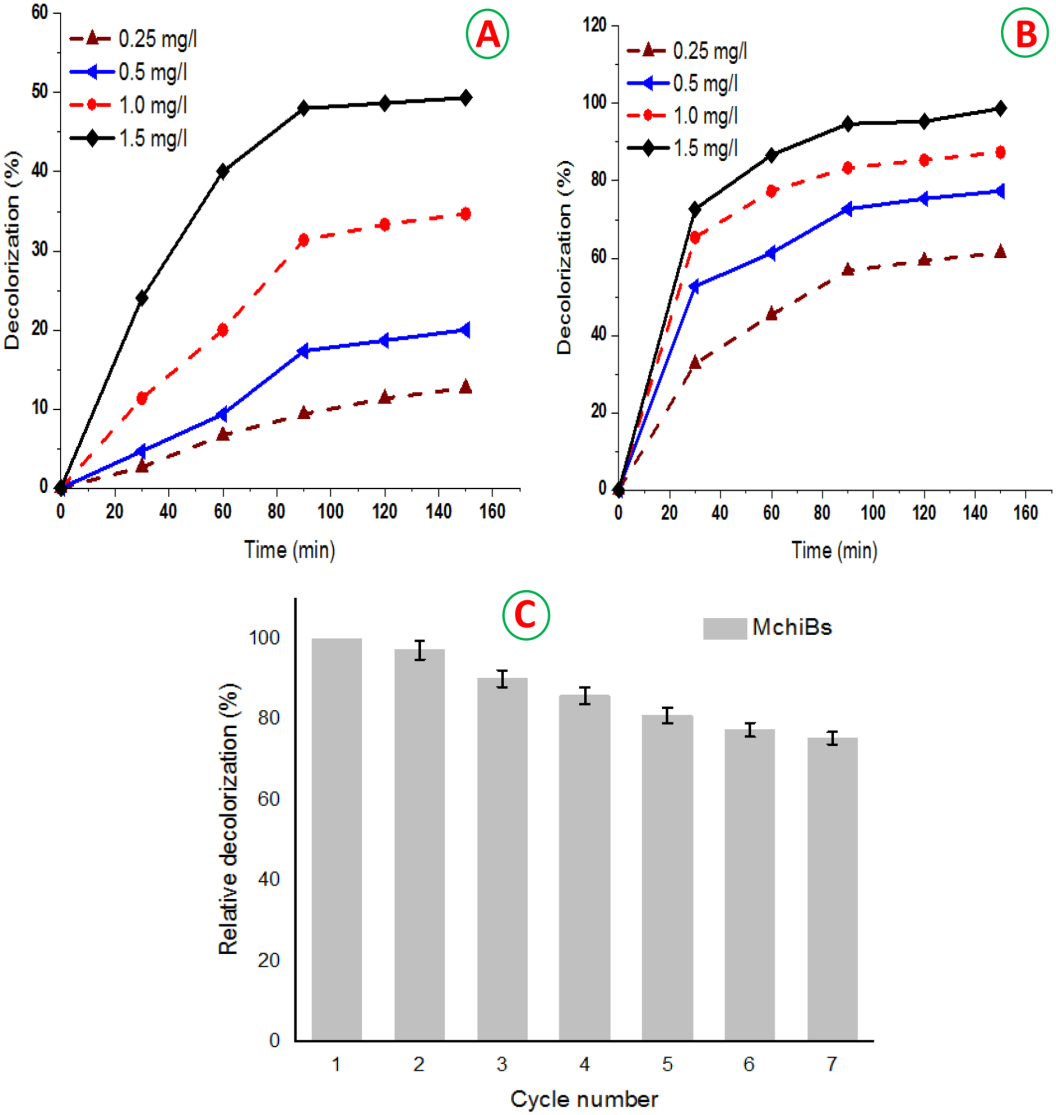
Decolorization percentage of real textile wastewater treated with control chitosan-beads (**A**) and magnetic-chitosan gel beads (**B**) using various concentrations (0.25–1.5 g/l) at different time intervals (30–150 min) under the same incubation conditions. (**C**) Relative decolorization percentage of the repeated treatment cycles using MchiBs. The setup performed under the optimal conditions. The washed beads were used for the subsequent cycles and the decolorization percentage was computed in relation to the 1st treatment cycle.

The efficiency of MchiBs for textile wastewater treatment investigated through batch experiments. The set up was carried out using various concentrations (0.25–1.5 g/l) at different time intervals (30–150 min) (Fig. 7A,B). The results reveal the decolorization percentage increased on continuing elevation in the MchiBs quantity from 0.25 to 1.5 g/l. The optimal removal percentage (94.7%) was determined at 90 min using 1.5 g/l of biosorbent (Fig. 7A). Whereas, the minimum decolorization percentage after 90 min incubation found to be 56.7% using 0.25 g/l of nanosorbent. The bioremediation process showed no remarkable difference when the process prolong for 150 min. The probable reason is the presence of more available sites for the uptake of dyes at the beginning of the batch experiment using biosorbent beads. The developed modified beads have a surface rich in the protonated –OH and –NH_2_ groups due to chitosan network. The sulfonic groups of dyes are vulnerable and show a low penetration ability in chitosan, which increase the accessibility of such groups to adsorption sites^1,13,36,45,46^.

The successful reusability of biosorbent for multiple cycles is very important parameter for the sustainability and potentiality of biosorbent in the bioremediation processes. Therein, the decolorization potentiality of MchiBs for seven repeated treatment cycles was assessed using textile wastewater at the optimal sorbent concentration and the optimal incubation time. The decolorization efficiency using MchiBs displayed 97.18, 91.14, 85.91, 80.98, 77.46 and 75.35% during successive decolorization trials for textile wastewater (Fig. 7C). After the 7th cycle, 75.35% decolorization was determined^5^ reported the enhancement of dye removal efficiency by using magnetic chitosan beads. One of the most important features of the magnetic chitosan beads is the ease retrieve from treatment solution and use for the consequent contaminants removal^35,47,48^.

The treatment process of textile wastewater using MchiBs was also evaluated through determination of pH, TDS, TSS, COD, EC, and PO_4_ as compared to untreated samples (control) under the same conditions (Table 3a). The treatment process conducted using 1.5 g/l biosorbent dose for 90 min. The results showed a highly significant reduction of TDS and COD from 1471.64 to 492.67 mg/l and from 1062.1 to 75.67 mg/l, respectively. In addition, the MchiBs could respectively reduce TSS EC, and PO_4_, related to the control textile wastewater samples. The removal percentage of textile wastewater contaminants using MgO-NPs was 72.2, and 92.1% for TDS, and COD. In addition, Fe_2_O_3_-NPs displayed 47.6, and 82.8%, respectively; however, the other tested parameters were considerably reduced, compared to untreated samples^2,41^. The polyaluminum chloride exhibited a reduction percentage of 84% for TDS^49^. The removal (%) of the investigated parameters using MchiBs exhibited remarkable improvements (94.88, 92.91, and 92.23%) for TDS, COD, and PO_4_, respectively, when compared with other latest nanosorbent (Table S2).

**Table 3:** Treatment of textile and industrial wastewater with magnetic-chitosan gel beads at concentration of 1.5 g/l and 90 min of incubation period.

Parameter	Untreated	Treated	Paired *t* test	
			*t*	*P* value
**a. Textile wastewater**				
pH	7.14 ± 0.14	8.63 ± 0.25	6.92	< 0.001*
TSS (mg/l)	1471.67 ± 6.51	492.67 ± 4.73	370.03	< 0.001*
TDS (mg/l)	684.67 ± 4.50	35.33 ± 3.51	168.91	< 0.001*
COD (mg/l)	1062.1 ± 9.64	75.67 ± 15.37	296.80	< 0.001*
EC (µmhos)	1387.33 ± 8.50	653.33 ± 18.58	47.35	< 0.001*
PO_4_ (mg/l)	32.67 ± 2.51	2.54 ± 1.41	46.93	< 0.001*
**b. Industrial wastewater**				
pH	7.47 ± 0.11	8.66 ± 0.45	20.95	0.002*
TSS (mg/l)	853.67 ± 7.02	492.33 ± 6.51	46.35	< 0.001*
TDS (mg/l)	552.67 ± 9.45	54.67 ± 3.51	172.71	< 0.001*
COD (mg/l)	1187.33 ± 6.98	234.33 ± 3.52	106.33	< 0.001*
EC (µmhos)	1069.67 ± 14.01	513.67 ± 4.73	52.46	< 0.001*
PO_4_ (mg/l)	85.34 ± 3.05	5.12 ± 0.07	46.42	< 0.001*

### Treatment of industrial wastewater using MchiBs

The physicochemical parameters of industrial effluent exhibited significant reduction of TSS, COD, and PO_4_ due to MchiBs treatment with 92.73%, 80.26% 94.0% and 88.19%, respectively (Table 3b). Meanwhile, the TDS and EC remarkably reduced by percentages of 42.33% and 51.98%, respectively. On contrary, the pH value was increased (pH 8.66).

The real industrial and textile wastewater have high organic contents with non-degradable nature, which are lost during dyeing process in the effluent^37,45^. This may attributed to the high value of various physicochemical features (pH, TDS, TSS, COD, EC, and PO_4_) of untreated effluents^2,50^. The removal of contaminants (heavy metals) using the Fe_3_O_4_-NPs incorporated into chitosan and regeneration of beads was reported by^1,4,5^. The physicochemical characters of textile wastewater remarkable reduced by treating with the nanoparticles, particularly COD, as determined by other researchers^37,51^. Similar results have reported for the significant reduction of industrial and textile wastewater parameters when treated with various biosorbent systems^4,36,37^.

## Materials and methods

**Figure 8:**
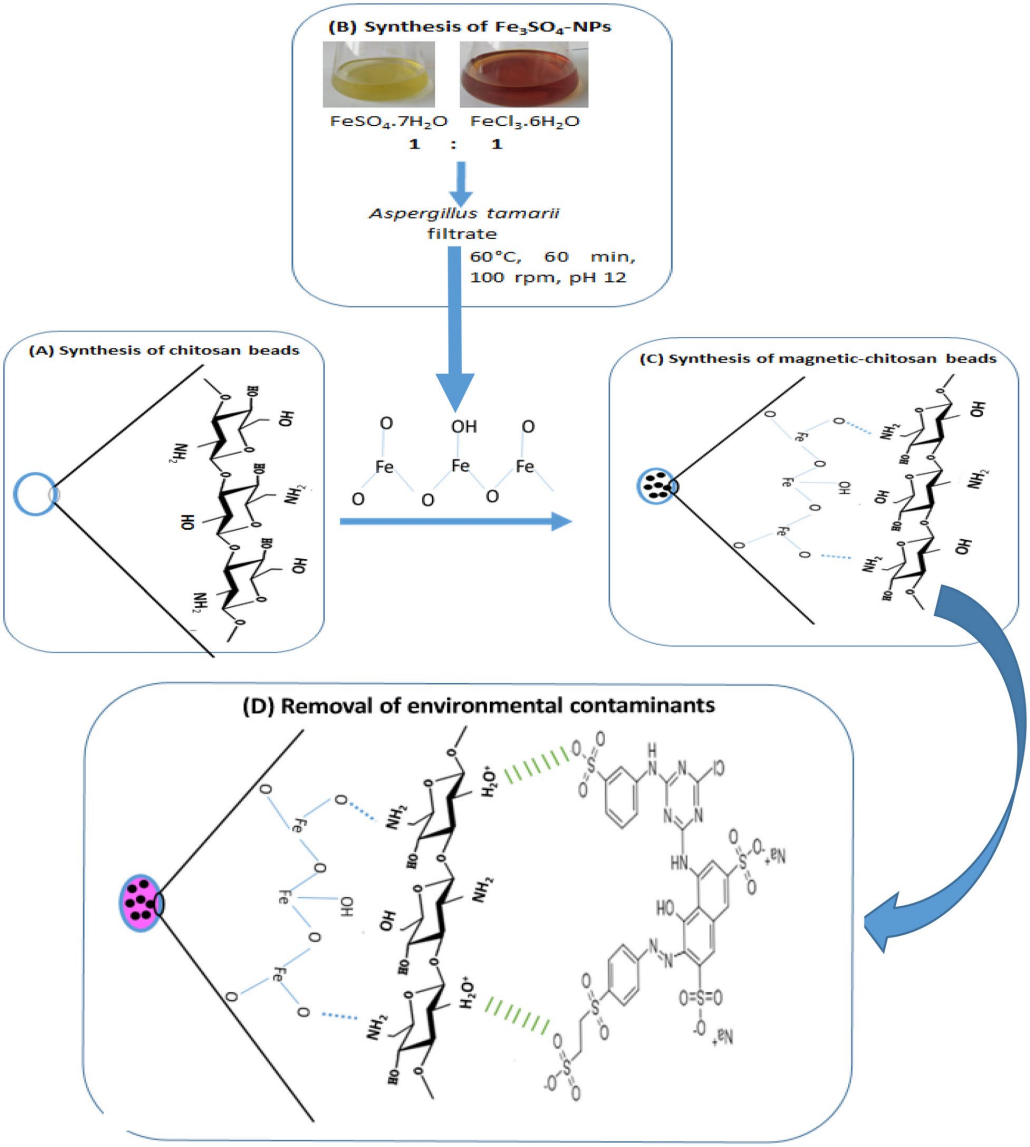
The schematic illustration of the synthesis of (**A**) control chitosan beads, (**B**) Fe_3_O_4_-NPs, (**C**) magnetic-chitosan nanocomposite with proposed surface and (**D**) the adsorption possibilities of the reactive red dye as pollutant model.

The overall process for the synthesis of control chitosan beads (CchiBs), magnetic chitosan beads (MchiBs), and the proposed adsorption mechanism for a dye pollutant model clearly shown in Fig. 8.

### Materials

Ferrous sulfate (FeSO_4_.7H_2_O, 99%) and Ferric chloride (FeCl_3_.6H_2_O, 97%) were of analytical grade and employed for the biosynthesis of magnetic nanoparticles. Chitosan and other chemicals used in the current research investigation purchased from Sigma-Aldrich (Cairo, Egypt) and were of analytical grade. The textile samples and industrial wastewater were collected from Egyptian factories, Egypt. These factories utilize different dyes like reactive red 198, reactive red 195, and reactive red 141 and organic acids (formic and acetic acid).

### Fungal strain

The fungal strain EG-MO7, which was previously isolated from a soil sample (Benha, Egypt), was employed in the current study for the synthesis of magnetic nanoparticle. In brief, the isolation was performed by inoculating 1.0 ml of 10^–5^ soil dilution onto Czapek’s-Dox agar (CDA) medium. The plate was incubated at 30 °C for 5 days. The developed purified strain was preserved on CDA medium slant for further study^52^.

### Morphological and molecular identification of fungal strain

The fungal isolate was recognized according to its morphological and macroscopically features using standard key of the genera *Aspergillus* sp.^53,54^. Such primary identification of fungal strain was affirmed by sequencing its internal transcribed spacer (ITS-rDNA) segment^28,55^. The fungal genomic DNA was extracted according to^56^. In the PCR, the gDNA was employed as template for the primers of ITS1 (5’-TCC GTA GGT GAA CCT GCG G-3’) and ITS4 (5’-TCC TCC GCT TAT TGA TAT GC-3’) using 2 × PCR master mixture (AlphaDNA Co, Canada). The PCR was performed in a Solgent EF-Taq, PCR Machine name: 9700(ABI), MJ research thermal cycler (USA). PCR amplification was conducted for 3 min at 95 °C, followed by 35 cycles for 30 s at 95 °C, 50 °C for 30 s and 72 °C for 90 s and next a final extension for 5 min at 72 °C. The PCR amplicon was investigated throughout 1% agarose gel electrophoresis, and then was sequenced by the same primer sets. The nucleotide sequence obtained from the ITS sequence was related to the ITS sequences in the GenBank database. Multiple sequence alignment were performed using ClustalW muscle algorithm of MEGA-X 11 and a phylogenetic tree was conducted by applying the neighbor-joining method with 1000 bootstrap analysis.

### Green synthesis of Fe_3_O_4_-NPs

The capability of *A. tamari* EG-MO7-exometabolites in the biosynthesis of Fe_3_O_4_-NPs was investigated. Briefly, a plague (1 cm) of the fungal culture of *A. tamari* (10^6^ spore/ml) was inoculated into 100 ml of CDA broth medium, containing % (yeast extracts 0.06; Glucose 1.0; (NH4)2 SO4; K2HPO4 0.2; 0.1; MgSO4.7H2O 0.01; KH2PO4 0.07; pH 6.5) and incubated at 30 °C for 5 days on rotary shaker (150 rpm). The fungal pellets were harvested by filtration using Whatman filter paper No.1. The biomass filtrate was centrifuged for 20 min at 10,000 × g under 4 °C. The developed cell-free filtrate was then harnessed for the biosynthesis of magnetic nanoparticles.

Magnetite nanoparticles were indigenously synthesized using the mixture of FeSO_4_.7H_2_O and FeCl_3_.6H_2_O in 1:1 ratio^2,4,28^. The metal solution was mixed with 50 ml of fungal supernatant, incubated at ambient temperature in dark conditions for 24 h. The preparation was heated at 60 °C, stirred for 1 h at 100 rpm and the pH was retained at pH 12. The resulted intense black color was monitored using UV/visible spectrophotometer. Subsequently, the preparation was kept at lab temperature for 1 h and then settled by centrifugation at 5000 × g for 20 min, and washed several times by distilled H_2_O. The as-formed Fe_3_O_4_-NPs were oven-dried at 60 °C for 12 h.

### Optimization of Fe_3_O_4_-NPs production using two-factorial Plackett–Burman design

In order to investigate the influences of different significant variables in Fe_3_O_4_-NPs production, five selected main variables namely incubation period (X_1_), temperature (X_2_), pH (X_3_), stirring speed (X_4_) and stirring time (X_5_), were analyzed using Plackett–Burman design (PBD) as illustrated in Table S3. The investigated variables were selected based on literature reports on the Fe_3_O_4_-NPs production^26,27^. Each independent variable was checked at 2-levels namely: high level (+1) and low level (−1), based on the fact that the biosynthesis of nanoparticles is remarkably regulated by the physicochemical parameters^10,26,57^. PBD depends on a 1st order model: Y = β_0_ + ∑β_i_ X_i_ where, Y was the height of absorbance peaks (response), β_0_ and β_i_ were the constant coefficients and X_i_ was an independent variable. The experiments were conducted in duplicate and the process optimization was investigated by spectrophotometrically measuring the height of absorbance peaks at the previously detected surface plasmon resonance^2^. Analysis of variance (ANOVA) was employed for testing the significance of each variable in the model. The investigated variables which exhibiting the major positive effects were chosen according to the results of Pareto chart, followed by further optimization throughout the Central composite design (CCD) of Response Surface Methodology. Further, three selected independent variables were optimized using CCD with α value of ± 1.681. The correlation between the response data and the variables were analyzed using a second order polynomial equation. 2D response plots were plotted, to investigate the major effects as well as the interactive ones between the dependent variable (response) and the independent ones.

### Synthesis of magnetic-chitosan gel beads (MchiBs)

The production of magnetic-chitosan gel beads was performed according to^1,35^ via the modified sol–gel method (Fig. S3). In brief, a desirable amounts of chitosan powder was dissolved using magnetic stirring for 15 min in 50 ml of CH_3_COOH 1% (v/v). 0.15 g of Fe_3_O_4_-NPs were added into the previous solution with magnetic stirring for 4 h. The resultant homogenous blend was added dropwise into NaOH 10% (v/v). Spherical magnetic-chitosan beads were developed and then kept for 24 h in NaOH. Such MchiBs were washed thrice using distilled H_2_O, until the neutrality of the wash off was obtained. Finally, the beads were dried at 50 °C for 12 h. By applying the same procedure without adding Fe_3_O_4_-NPs, a control chitosan beads (CchiBs) were also prepared^5^.

### Characterization

The size and shape of the biosynthesized NPs were obtained using JEOL JEM-1010 transmission electron microscope (TEM, Japan). The samples were drop coated on carbon grid for TEM analysis and voltage was retained at 100 kV. The functional group pattern was assessed by Fourier transform infrared (FTIR) spectroscopy. All samples were examined within a range of 450–4000 cm^-1^. The morphological characterization of CchiBs and MchiBs were carried out by scanning electron microscope (SEM) (JSM-6510LV microscope, JEOL, Tokyo, Japan), which operated at 10 keV. The elemental analysis of Fe_3_O_4_-NPs was determined using energy-dispersive X-ray detector, which attached to JEOL JSM-6510LV SEM at 10 keV. Finally, the crystalline structure of the magnetic nanomaterial was confirmed by X-ray powder diffraction (XRD), X’PERT PRO, (MiniFlex 300/600 X-ray, USA) which operated at 28 °C and 40 kV with a radiation source of Cu-Ka. The samples of Fe_3_O_4_-NPs, CchiBs and MchiBs were scanned in the range of 2θ from 10 to 90° at 10.00° min scanning rate.

### Applications of CchiBs and MchiBs in textile wastewater treatment

The capability of control chitosan beads (CchiBs), and magnetic chitosan beads (MchiBs) for textile wastewater treatment was performed in batch experiments. A known amount of CchiBs and MchiBs (0.25, 0.5, 0.1, 1.5 g/l) was added to wastewater samples. The systems were incubated in dark at ambient temperature with continuous agitation to attain proper oxygenation. The decolorization efficiency was determined at various time intervals (30, 60, 90, 120 and 150 min) by withdrawing 1 ml of each treatment, centrifuged for 15 min at 5,000×g, and the residual concentration of the dye was monitored at λ_max_ = 530 nm using UV–visible. The decolorization percentage (D.P., %) was calculated using the following equation^28,58^:

2 $${\text{D}}.{\text{P}}.\left( \% \right) = \frac{{C_{i} - C_{f} }}{{C_{i} }} \times 100$$

where ${C}_{i}$ and ${C}_{f}$ are the initial and final dye concentration.

The reusability of the investigated MchiBs was assessed for textile wastewater treatment through seven consecutive cycles under the optimal conditions according to^28,52,59^ with some modification. Afterward the completion of the 1st biosorption cycle as declared above, the MchiBs were separated using external magnet, next rinsed many times with distilled water and then used for the next cycle. The relative decolorization percentages were calculated, related to the first degradation cycle^28,52^.

The physicochemical parameters; total dissolved solids (TDS), pH, total soluble salts (TSS), chemical oxygen demand (COD), conductivity (EC), phosphate and sulfur were determined before and after the treatment with MchiBs according to^60^ at the optimal time of decolorization and biosorbent concentration.

### Industrial wastewater treatment using MchiBs

The batch experiments were conducted using MchiBs at the optimal concentration and the best incubation time for treatment of industrial wastewater. The physicochemical parameters (pH, TDS, TSS, COD, EC, and PO_4_) were measured according to the standard protocols^60^.

### Deposition of the fungal strain

The fungal strain *A. tamari*, was deposited into the GenBank under accession number OL824549.1.

### Data statistical analysis

All experiments were carried out in triplicates and data was articulated as mean ± standard deviation. The process of nanoparticle production was optimized by screening the selected independent variables using the Placket–Burman design. The analysis was conducted using MINITAB 18.0 statistical software package, USA. In order to explore significant differences at a confidence level of 95% (*P* < 0.05), the paired *t* test was carried out. Statistical Package for Social Sciences (SPSS) version 25 (IBM, Armonl, Ny, USA) was used for conducting the data processing statistical analysis.

## Conclusions

The successful biosynthesis of Fe_3_O_4_-NPs by harnessing the exo-metabolites present in the fungal filtrate of *A. tamarii* EG-MO7 was performed. Based on PBD and CCD, the optimum magnetic nanoparticle production was determined with the contribution of incubation period (24 h), temperature (30 °C), pH (12), stirring speed (100 rpm) and stirring time (1 h); however, the highest effect was recorded for the pH. The Fe_3_O_4_-NPs impregnation into chitosan beads, which was successfully, performed using sol–gel method. The modified beads exhibited a remarkable decolorization rate with considerable regeneration property and a highly significant reduction of various physico-chemical parameters (pH, TDS, TSS, COD, and PO_4_) in short treatment period (90 min). The exact mechanism of the fungal exometabolites incorporate in the nanoparticle synthesis should be performed in further studies. The performance of Fe_3_O_4_-NPs-based polymer in the bioremediation process was found to be simple, satisfactory, and perform potential advantages in the bioremediation of wastewater contaminants. However, the detailed adsorption mechanism, kinetics and isotherms of the modified beads for the dye removal should be performed in future studies. Large-scale production of the above-mentioned chitosan composite, especially in the developing countries, required further research for reducing the biosynthetic cost. The formation of secondary pollutants due to the usage of chitosan composite in bioremediation processes is a major drawback during the recycling and regeneration of chitosan nanocomposite.

## Supplementary Information

Supplementary Information.

## Data Availability

All data generated or analyzed during this study are included in this article and its additional file.

## Abbreviations

NPs: Nanoparticles
PBD: Plackett–Burman design
CCD: Central composite design
RSM: Response surface methodology
BOD: Biological oxygen demand
COD: Chemical oxygen demand
TDS: Total dissolved solids
TSS: Total soluble salts
EC: Conductivity
CchiBs: Control chitosan beads
MchiBs: Magnetic-chitosan gel beads
TEM: Transmission electron microscope
SEM: Scanning electron microscope
EDX: Energy-dispersive X-ray detector
XRD: X-ray powder diffraction
FTIR: Fourier transform infrared spectroscopy
D.P.: Decolorization percentage
ANOVA: Analysis of variance
SPSS: Statistical package for social sciences
