# Supplementary material for: Aspergillus tamarii mediated green synthesis of magnetic chitosan beads for sustainable remediation of wastewater contaminants
Source: Sci Rep. 2022 Jun 13;12:9742. doi: 10.1038/s41598-022-13534-1 (PMC9192714; doi:10.1038/s41598-022-13534-1)
Supplement: Supplementary file 1 — Supplementary Information. [file 41598_2022_13534_MOESM1_ESM.pdf]

Scientific Reports

Aspergillus tamarii mediated green synthesis of magnetic chitosan beads for sustainable remediation of wastewater contaminants

Reyad M. El-Sharkawy*, Mahmoud A. Swelim & Ghada B. Hamdy

Botany and Microbiology Department, Faculty of Science, Benha University, Benha 13511, Egypt;

* Correspondence: r.m.elsharkawy@fsc.bu.edu.eg, https://orcid.org/0000-0003-1319-9066

Author information

Affiliations

Botany and Microbiology Department, Faculty of Science, Benha University, Benha 13511, Egypt;

Reyad M. El-Sharkawy, Mahmoud A. Swelim & Ghada B. Hamdy

Corresponding authors

Correspondence to Reyad M. El-Sharkawy, email**:**

r.m.elsharkawy@fsc.bu.edu.eg, https://orcid.org/0000-0003-1319-9066

Table S1: Generated CCD matrix for independent variables used RSM

Trials	Variables			Absorbance	
	X_1.Incubation period	X_3. pH	X_5.Stirring time	Actual	Predicted
1	24	5	1	1.63	1.51
2	48	5	1	1.05	0.66
3	24	12	1	3.68	3.15
4	48	12	1	2.08	1.74
5	24	5	2	0.92	0.97
6	48	5	2	0.57	0.82
7	24	12	2	1.16	1.27
8	48	12	2	0.72	0.56
9	15	8.5	1.5	1.87	2.02
10	56	8.5	1.5	0.46	0.71
11	36	2.6	1.5	0.48	0.47
12	36	14.3	1.5	1.22	1.63
13	36	8.5	0.7	1.81	2.49
14	36	8.5	2.3	1.32	1.04
15	36	8.5	1.5	2.15	2.33
16	36	8.5	1.5	2.28	2.33
17	36	8.5	1.5	2.37	2.33
18	36	8.5	1.5	2.23	2.33
19	36	8.5	1.5	2.41	2.33
20	36	8.5	1.5	2.61	2.33

Table S 2: Comparison of different parameters of textile and industrial wastewater treated with MchiBs with other nanosorbent reported in the literature.

Biosorbent		Removal (%)							References
		TSS	TDS	COD	EC	PO_4	Decolorization (%)	Time (min)	
Fe_3O_4/Chitosan	Textile wastewater	66.52	94.88	92.91	52.91	92.23	94.7	90	**PS**
	Industrial wastewater	42.33	90.11	25.1	51.9	94.0	-	-	
MgO-NPs		85.5	72.2	92.1	71.3	-	80.07	240	[37]
Fe_2O_3-NPs		75.7	47.6	82.8	46.9	-	74.2	240	[37]
PAC		78.05	84	-	80.85	-	85.0	30	[49]
Fe_3O_4-NPs		-	-	66.7	-	-	-	-	[46]
MgO-NPs		97.9	98.2	97.3	-	-	-	-	[8]
Fe_3O_4/3-MPA		-	-	-	-	-	45.3	100	[27]

PS: Present study, PAC: polyaluminum chloride, 3-MPA: 3-mercaptoproionic acid

Table S 3: Twelve run Plackett-Burman design for assessment of five independent variables. The optimization was determined by the height of absorbance peaks. "+1" and "-1" describe the high and low level of the tested parameter range.

**Run**	**Variables**					**Y**
	**X1**	**X2**	**X3**	**X4**	**X5**	**(Absorbance peak, a.u.)**
1	+1	-1	-1	-1	+1	0.69
2	-1	+1	+1	-1	+1	3.61
3	+1	-1	+1	-1	-1	3.22
4	-1	-1	+1	+1	+1	3.55
5	-1	+1	+1	+1	-1	3.21
6	+1	+1	-1	+1	-1	2.06
7	+1	-1	+1	+1	-1	2.98
8	-1	-1	-1	+1	+1	1.98
9	+1	+1	-1	+1	+1	1.34
10	-1	+1	-1	-1	-1	3.03
11	-1	-1	-1	-1	-1	3.09
12	+1	+1	+1	-1	+1	2.06

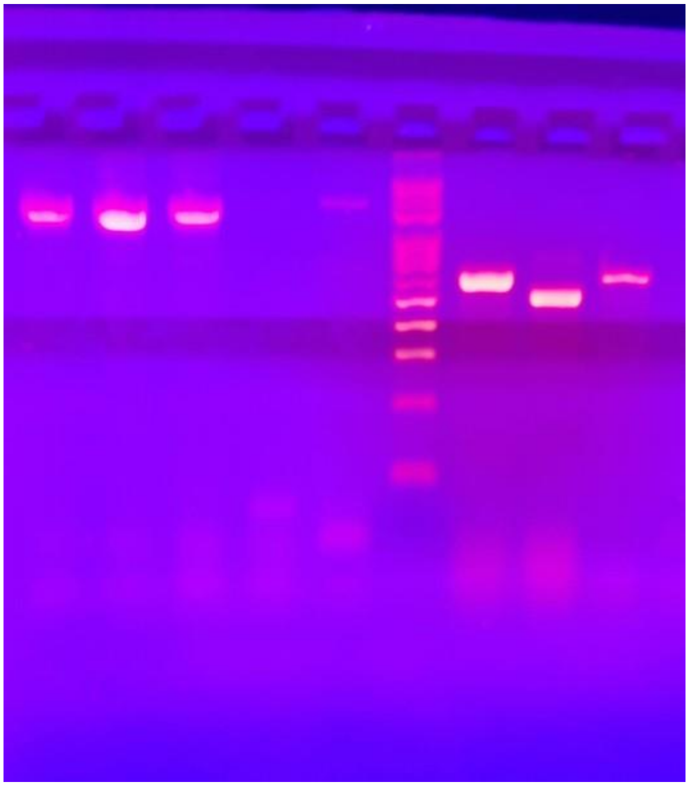

Fig. S1 Uncropped gel image of PCR product in Figure 1.

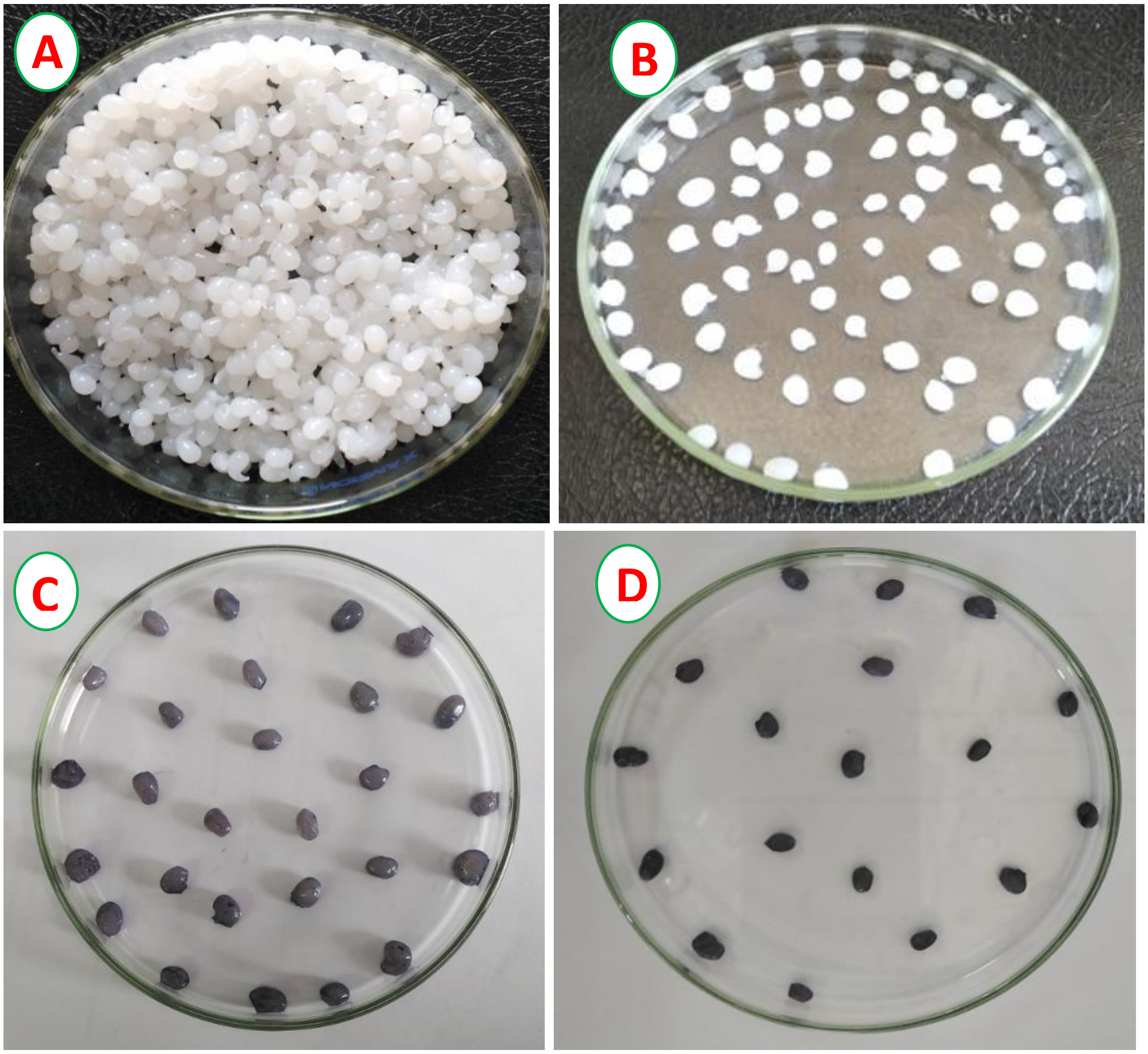

Fig. S2 Photograph images of wet (**A**) CchiBs, (**B**) dry CchiBs, (**C**) wet MchiBs, and (**D**) dry MchiBs.

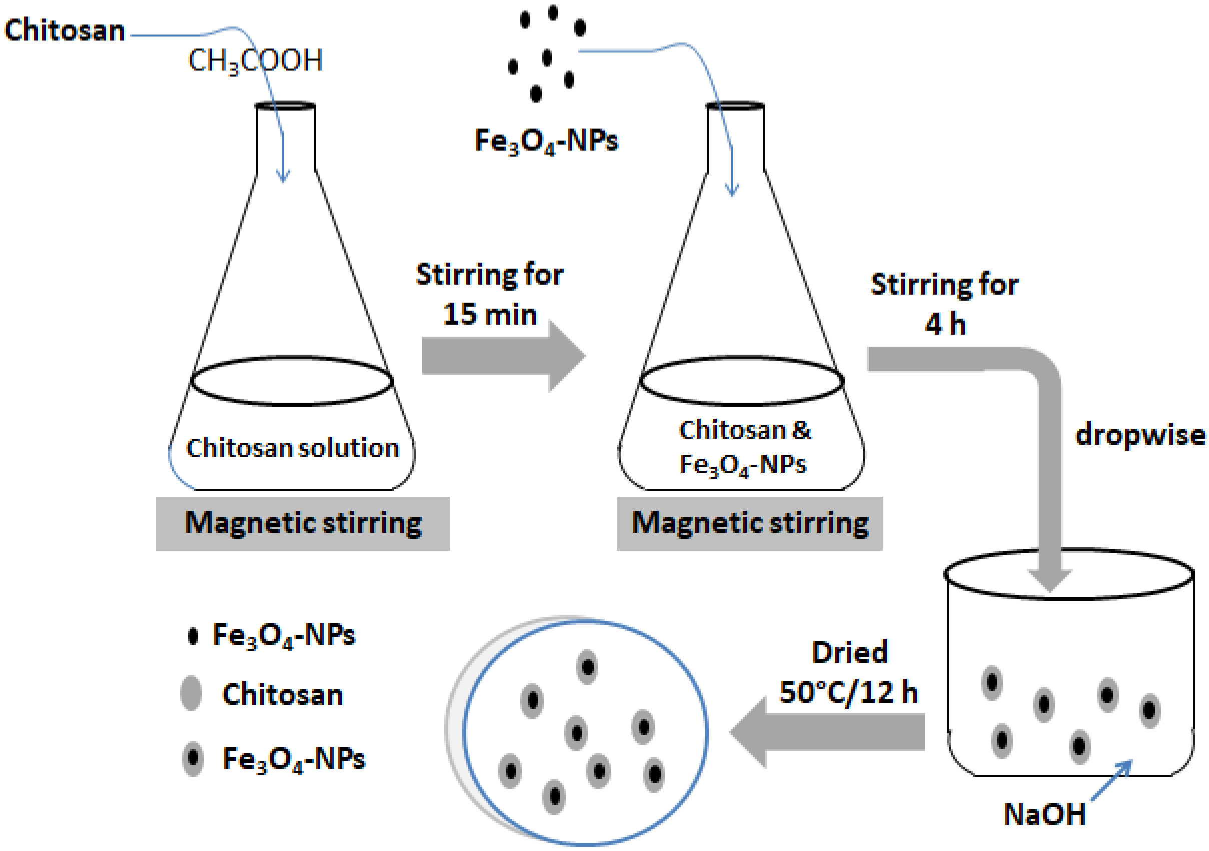

Fig. S3 Schematic diagram illustrating the synthetic process of magnetic-chitosan beads.
